# Wide Spectrum of Bradyarrhythmias and Supraventricular Tachyarrhythmias in Sportsmen: Run Forrest, Run?!

**DOI:** 10.31083/j.rcm2506221

**Published:** 2024-06-19

**Authors:** Zofia Kampka, Mateusz Drabczyk, Nina Piłka, Michał Orszulak, Maciej Rycyk, Katarzyna Mizia-Stec, Maciej T. Wybraniec

**Affiliations:** ^1^First Department of Cardiology, School of Medicine in Katowice, Medical University of Silesia, 40-635 Katowice, Poland; ^2^First Department of Cardiology, Upper-Silesian Medical Center, 40-635 Katowice, Poland; ^3^Abbott Electrophysiology, 02-676 Warsaw, Poland; ^4^Member of the European Reference Network on Heart Diseases - ERN GUARD-HEART, 1105 AZ Amsterdam, Netherlands

**Keywords:** bradyarrhythmia, tachyarrhythmia, exercise-induced arrhythmia, sports cardiology

## Abstract

The intricate relationship between sports participation and cardiac arrhythmias is a key focus of cardiovascular research. Physical activity, integral to preventing atherosclerotic cardiovascular disease, induces structural, functional, and electrical changes in the heart, potentially triggering arrhythmias, particularly atrial fibrillation (AF). Despite the cardiovascular benefits, the optimal exercise amount remains unclear, revealing a J-shaped association between AF and exercise. Endurance athletes, particularly males, face elevated AF risks, influenced by age. Risk factors vary among sports modalities, with unique physiological responses in swim training potentially elevating AF risk. Clinical management of AF in athletes necessitates a delicate balance between rhythm control, rate control, and anticoagulation therapy. Sport-induced bradyarrhythmias, including sinus bradycardia and conduction disturbances, are prevalent among athletes. Managing bradycardia in athletes proves challenging due to its complex and not fully understood pathophysiology. Careful consideration is required, particularly in symptomatic cases, where pacemaker implantation may be necessary for sinus node dysfunction. Although pacing is recommended for specific atrioventricular (AV) blocks, milder forms often prevail without restricting sports participation. This review explores the nuanced relationship between exercise and tachy- and bradyarrhythmia in athletes, addressing the challenges clinicians face when optimizing patient care in this distinctive population.

## 1. Introduction

In recent years, the intricate relationship between sports participation and cardiac arrhythmias has emerged as a focal point of cardiovascular research. Beyond any doubt, physical activity represents a cornerstone of primary and secondary prevention of atherosclerotic cardiovascular disease (ASCVD) and its complications. Physical activity can be both a preventive and a triggering factor for arrhythmias, with atrial fibrillation (AF) being the most common in both the athletes and general population. The sophisticated interplay between exercise and cardiovascular health is further complicated by physiological adaptations associated with intense physical training in competitive athletes. The modified expression of arrhythmias in this group of patients necessitates a comprehensive understanding of these phenomena. This article delves into the intricate interplay between exercise and sport-induced brady- and tachyarrhythmias, unraveling the complexities of its clinical management in athletes.

## 2. Influence of Sport on Heart

**Fig. 1.**
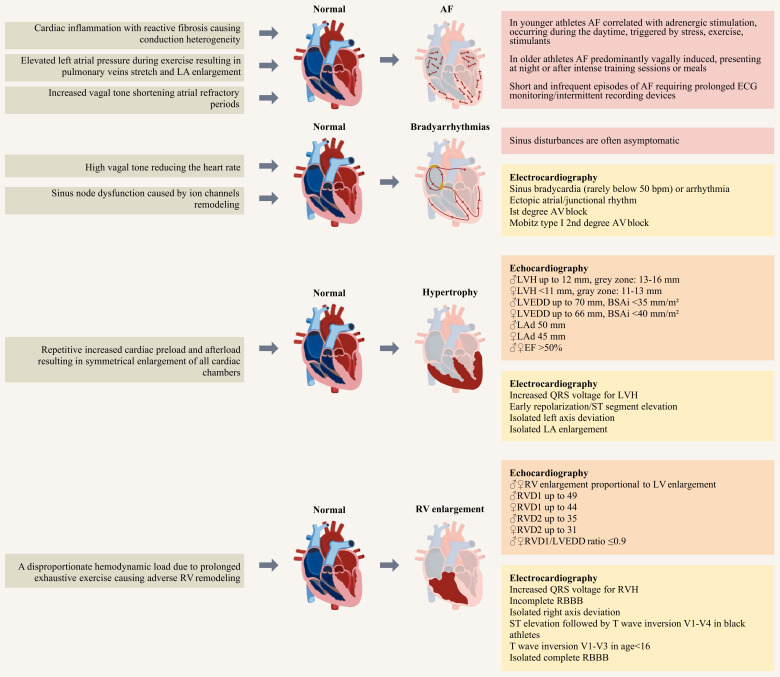
**The long-term effects of athletic training on electrical and structural cardiac remodeling [1, 2, 3, 4]. **This schematic illustration delineates the principal features and criteria linked to the electrical and structural adaptations in the heart induced by athletic training. The athlete’s electrocardiogram and echocardiogram exhibit a spectrum of variations influenced by factors including age, gender, and ethnicity. These variations may pose diagnostic challenges in distinguishing the athlete’s heart from cardiomyopathy. AF, atrial fibrillation; ECG, electrocardiogram; bpm, beats per minute; AV block, atrioventricular block; LVH, left ventricular hypertrophy; LVEDD, left ventricular end-diastolic diameter; BSAi, indexed to body surface area; LAd, left atrium diameter; EF, ejection fraction; LA, left atrium; RV, right ventricle; LV, left ventricle; RVD1, right ventricular basal diameter at end-diastole; RVD2, right ventricular mid diameter at end-diastole; RVH, right ventricular hypertrophy; RBBB, right bundle branch block.

The long-term effects of consistent endurance training can lead to multifaceted changes in cardiac structure, function, and electrical activity, which are summed up on Fig. 1 (Ref. [1, 2, 3, 4]). The above-mentioned process can blur the line between the athletic heart phenotype and certain cardiac pathologies, creating a diagnostic challenge known as the “grey-zone” [5].

Exercise-induced cardiac remodeling (EICR) occurs due to the pressure and volume stressors associated with heightened external and internal work, initiating adaptive changes in the heart. Endurance sports such as long-distance running, Nordic skiing, rowing, and cycling predominantly involve isotonic stress, involving the circulation of large blood volumes throughout the cardiovascular system. The repetitive application of isotonic stress over an extended duration typically leads to biventricular dilation, left and right atrial dilation, and enhanced left ventricular diastolic function. On the contrary, sports demanding brief, intense, repetitive bursts like power weightlifting, American football line play, and martial arts result in robust isometric stress, causing transient systemic blood pressure increase, left ventricle remodeling, and mild concentric hypertrophy. Numerous popular sports entail a substantial combination of isotonic and isometric cardiovascular stress [6]. Uneven wall stress, particularly affecting the thinner-walled right ventricular (RV) can potentially lead to asymmetry in remodeling [5].

**Fig. 2.**
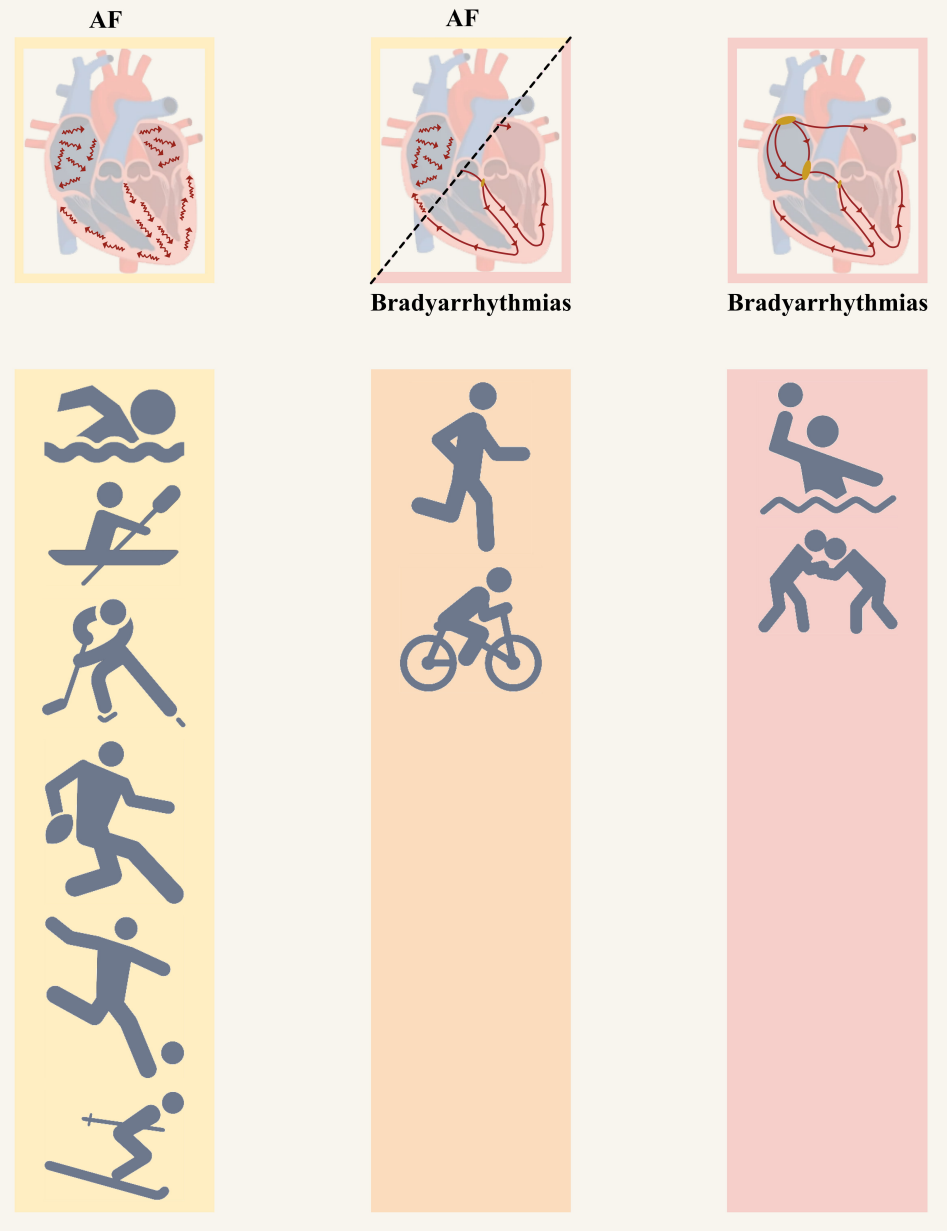
**Sport disciplines with the highest risk of tachy- and bradyarrhythmia in athletes [7, 8, 9, 10, 11, 12].** AF, atrial fibrillation.

The electrical effects of athletic training can be divided into two main categories: those influenced by high vagal tone and those indicative of enlarged cardiac chambers. Common electrocardiogram (ECG) patterns include sinus bradycardia, sinus arrhythmia, J-point elevation, first-degree atrioventricular (AV) block, voltage criteria for left ventricular (LV) and RV hypertrophy, and left and right atrial enlargement. Some athletes may exhibit nodal rhythm or Mobitz type 1 second degree AV block at rest, resolving with mild exertion. EICR is a proarrhythmic condition, posing a risk for AF, sinus node dysfunction, second-degree or third-degree AV block, and ventricular arrhythmias (Fig. 2, Ref. [7, 8, 9, 10, 11, 12]) [6, 13, 14].

Some sport disciplines predispose to AF and/or bradyarrhythmia more than others. The underlying physiological mechanisms are particularly expressed in endurance sportsmen. Adverse cardiac remodeling, fibrosis of the conduction system, vagal hypertonia and intrinsic heart changes, particularly eccentric and concentric hypertrophy of the left ventricle are the reasons for bradyarrhythmia in sportsmen. Increased vagal tone, microtrauma, inflammation and fibrosis accompanied by left atrial enlargement lead to atrial fibrillation.

## 3. The Paradox: AF and Physical Activity

Physical inactivity constitutes a modifiable, lifestyle-based risk factor for AF. Meeting the recommended levels of physical activity, specifically 150 minutes of moderate-intensity exercise per week, is associated with a diminished risk of developing paroxysmal AF. Exercise plays a pivotal role in managing established risk factors for AF, including obesity, hypertension, and diabetes, while concurrently contributing to the reduction of inflammation and the preservation of autonomic balance. These combined effects collectively decrease the likelihood of AF onset [15].

Nevertheless, determining the optimal dosage of physical activity remains a challenge. Although current guidelines propose a recommended activity level of approximately 450–900 metabolic equivalent of task (MET)-minutes per week, patients may derive additional benefits from elevating their activity to 1000–1500 MET minutes per week. It is crucial to note that the majority of studies rely on self-reported physical activity, potentially introducing reporting bias. There is a pressing need for studies incorporating more objective measures to accurately report physical activity levels [16].

The relationship between AF and exercise is further complicated by a J-shaped association. Both professional and nonprofessional athletes face an increased risk of AF compared to the general population, when extending the exercise load beyond a certain threshold. In patients with an established diagnosis of AF, clinicians should make patients aware of the ambiguous impact of physical exercise, which generally decreases a patient’s cardiovascular risk, but may constitute a trigger of an AF episode. The potential negative impact of intense physical activity has raised concerns not only about the optimal amount of exercise but also about the most suitable clinical management of such patients [7, 17].

## 4. AF Risk Inherent in Sports

The risk of AF is estimated to be two- to five-fold greater in endurance athletes than in non-athletic individuals. Evidence indicates that AF is influenced by factors such as the intensity, duration, and type of exercise or sport [18]. The well-established dose–response relationship between exercise and AF risk is notable, with athletes diagnosed with AF demonstrating higher weekly training volumes, engaging in longer training sessions, and participating in slightly more sports events per year [7]. There is an increasing need for studies capable of identifying the most suitable type of sport, as well as determining the optimal and safe regular “dose” of exercise before the risk of developing AF becomes significant [19].

Both mixed and endurance exercises have been associated with an increased risk of atrial fibrillation [18]. While observational studies have widely represented endurance athletes such as runners, joggers, cyclists, and skiers, there has been less emphasis on non-aerobic disciplines like weightlifting [20]. Notably, after adjusting for lifetime exercise dose, swimming may confer a stronger predisposition to AF compared to running or cycling. The underlying cause remains unclear, but two hypotheses attempt to explain this phenomenon. Firstly, the horizontal position may contribute to orthostatic intolerance in swimmers and alterations in autonomic modulation, which are linked to the development of AF. Secondly, exposure to cold water elicits distinct physiological responses. Cold shock provokes sympathetic autonomic-mediated tachycardia, which could make swimmers more prone to developing arrhythmia [7].

While strenuous exercise increases the risk of AF in men, its influence on women seems to be far more sophisticated. Current data suggests that intense exercise has either a protective or neutral effect on the likelihood of AF development in women [20]. The underlying cause of this gender-based difference is not yet fully investigated, but possible explanations include gender differences in cardiac remodeling. Female athletes exhibit different patterns of structural and electrical remodeling; therefore, conclusions drawn from studies involving men cannot be directly extrapolated to female athletes [21]. The association between AF, exercise, and gender is further complicated by the likely lower cumulative exposure to exercise in females and the significant underrepresentation of female athletes in previous studies, reflecting the insufficient representation of females in endurance sports competitions [22].

Age is a notable factor influencing the incidence of AF in athletes. A significantly higher occurrence of AF has been observed among middle-aged athletes, with the heightened risk diminishing after the age of 55 [18, 20]. The underlying mechanisms also evolve with age. In younger athletes, the first AF incidents are typically correlated with adrenergic stimulation, occurring during the daytime and triggered by factors such as stress, exercise, or stimulants like caffeine. On the other hand, in older athletes, the arrhythmia is predominantly vagally induced, presenting at night or after intense training sessions or meals. Additionally, greater lean body mass and height are noteworthy predictors in the incidence of AF in athletes [17].

Many population-based studies reported racial differences in AF (the highest incidence in White populations) [23, 24]. Studies investigating the impact of ethnicity on AF burden in athletes are lacking, and so far racial disparities in this population remain unknown [25].

## 5. Screening for AF in Athletes 

Although endurance sports are well-established risk factors for AF, identifying at-risk athletes poses a challenge. Self-reported exercise training history, being a subjective indicator, often overlooks training intensity. Relying solely on left atrial enlargement as a predisposing factor for atrial arrhythmias is limited, given that left atrial dilatation is a common trait in athletes’ hearts, and the contribution of left atrial size to AF in athletes is debatable. The assessment of atrial fibrosis with cardiac magnetic resonance appears promising for identifying at-risk individuals; however, the significance of atrial fibrosis as a predictor of AF in athletes requires further investigation [17, 26].

Many athletes exhibit symptoms of exercise intolerance and acute fatigue during the onset of AF, while some remain entirely asymptomatic. Similar to non-athletes, the intriguing heterogeneity in symptomatology lacks a clear explanation [17]. Athletes typically experience short and infrequent episodes of AF, necessitating prolonged ECG monitoring or intermittent recording devices for accurate detection of paroxysmal AF [27]. Although AF in athletes is seldom caused by underlying structural heart disease or comorbidities, it is crucial to consider hidden risk factors such as hypertension, thyroid disease, alcohol consumption, performance-enhancing agents, and illicit drugs [17, 21].

Nowadays, there is an opulence of digital devices focused on heart rate monitoring. Those heart rate monitors (HRMs) use either electrocardiac sensors or photoplethysmography (PPG) technology to evaluate heart rate. The electrocardiac-based devices have superior performance but PPG devices are smaller, more easily worn, and lower cost which makes them more widespread. However, we must keep in mind that none of these devices is designated as a medical-graded HRM during exercise and abnormal HRM read-out should be evaluated by an experienced physician and confirmed by ECG recordings. In the European Heart Rhythm Association (EHRA) practical guide dealing with digital devices there is a diagnostic evaluation flowchart for athletes who present with abnormal HRM readings and/or suspected arrhythmias [28].

## 6. Specific Aspects of AF in Athletes

An episode of AF in athletes presents in a dual nature, exhibiting itself as either a poorly tolerated tachyarrhythmia or AF with a slow ventricular rate, leading to a decline in exercise tolerance. The high rate subtype of AF in athletes is characterized by rapid and irregular heartbeats, contributing to symptoms such as palpitations and chest discomfort. Conversely, the manifestation of AF with a slow ventricular rate suggests compromised cardiac output during episodes, leading to a decline in exercise tolerance. The presence of an excessively slow heart rate (below 50 to 60 beats/min) accompanying AF episodes hints at a potential underlying disease affecting the AV node. Regardless of the manifestation, both types of AF episode warrant consideration of pulmonary vein isolation (PVI). However, understanding of these variations is crucial, as they represent distinct stages of heart overload during physical exertion [29, 30].

When considering appropriate anticoagulation therapy, the CHA2DS2-VASc score may not serve as an optimal risk stratification method for athletes. Although the majority of athletes receive 0 or 1 point and are not typically offered anticoagulation, it is essential to recognize that AF itself is an independent risk factor for stroke [7]. Additionally, in young women without other risk factors, gender does not contribute to an elevated risk of stroke on the CHA2DS2-VASc score [31].

Notably, the diagnosis of AF in professional athletes does not seem to confer an increased risk of death. This phenomenon may be attributed to the beneficial effects of training, including the relatively low prevalence of other cardiovascular diseases, such as coronary heart disease, in this population [19].

The use of direct oral anticoagulant (DOAC) therapy can pose as a challenge for athletes, as it excludes them from participating in contact sports or disciplines with a high risk of trauma. Athletes requiring DOAC treatment may benefit from personalized timing of therapy, enabling them to engage in training and competitions, especially those involved in contact or high-impact sports [19]. Also, sportsmen should be made aware of the presence of reversal agents such as idarucizumab for dabigatran and andexanet-alfa for factor Xa inhibitors, which could decrease their fear of anticoagulation-related hemorrhagic complications [32]. However, athletes on DOAC therapy may express concerns about the risk of bleeding associated with potential injuries during sports activities, leading them to limit their training. This precautionary approach is consistent among participants engaged in both high-risk sports, such as mountain biking and cycling, and those involved in lower-risk sports [33].

## 7. Clinical Management 

For young and middle-aged athletes with recurrent exercise-induced symptomatic paroxysmal AF, reducing physical activity may be a strategy to mitigate AF triggers. However, the association between limiting physical activity and long-term outcomes remains unclear, and there is currently insufficient evidence to advocate for detraining as a broad recommendation for athletes with AF [34]. Adjusting to the newly imposed training restrictions poses a significant challenge for athletes, who express frustration over the substantial decrease in both the duration and intensity of their training [35].

In athletes, rhythm control is preferred over heart rate control. Athletes without structural heart disease may be offered the pill-in-pocket approach or regular treatment with flecainide, propafenone or sotalol. Also, in patients with vagally-mediated night-time or post-prandial AF, a class IA disopyramide may serve as an efficacious antiarrhythmic agent [33, 36]. While regular treatment is more effective, many athletes prefer to avoid daily medication. In rate-control therapy, beta-blockers are often poorly tolerated. Due to an increased risk of atrial flutter and atrial tachycardia with rapid ventricular response, successful therapy with flecainide or propafenone may involve adding a beta-blocker or non-dihydropyridine calcium channel blocker. It is crucial to note that amiodarone, with its long-term toxic effects, is not recommended for regular treatment in the young population without structural heart disease, including athletes [17, 19].

In general, drugs for rate and/or rhythm control can adversely impact athletes’ training abilities, leading to a conflict between achieving optimal medical management and sustaining their athletic lifestyle [35]. Athletes experiencing persistent symptomatic AF or those who do not respond well to or cannot tolerate medical therapy should consider AF ablation. PVI with radiofrequency (RF) ablation is a potentially successful therapy in athletes, providing freedom from AF recurrence. In selected athletes, PVI may even be considered a first-line treatment, taking into account athlete preferences and the type of sport they engage in [19, 26].

## 8. Bradyarrhythmia among Athletes

Sport-induced bradyarrhythmias, encompassing sinus bradycardia or conduction disturbances leading to a decreased heart rate, such as atrioventricular blocks (AVB), are generally regarded as non-pathological phenomena in athletes. This remains true unless they are accompanied by symptoms or coexist with other arrhythmias [37]. Bradycardia is defined as a heart rate below 60 beats per minute (bpm) [38]. A heart rate ranging from 51 to 60 bpm is commonly observed in athletes, while rates below 50 bpm are rare and are primarily noted in highly trained endurance athletes [8]. Notably, extreme forms of bradycardia, such as a resting sinus rhythm below 30 bpm, have only been reported in elite athletes during nocturnal periods. It is crucial to emphasize that the post-exertional increment in heart rate holds significance in the interpretation of bradycardia [39].

## 9. Pathophysiology and Underlying Mechanism 

Although bradycardia is commonly prevalent among sports athletes, its mechanism is not fully and clearly understood. Recent research provides evidence that the underlying mechanism of bradycardia may have a multifactorial origin [40].

## 10. Autonomic Regulation 

It is widely believed that bradycardia is caused by high vagal tone impacting the sinus node or atrioventricular node (e.g., inducing AVB), which results in a reduction of heart rate [39, 41]. The greater the vagal activity, the lower the recorded heart rate tends to be. However, the precise measurement of the physiological activity of the vagus nerve on the heart’s pacemaker is technically challenging due to the simultaneous conduction of efferent and afferent fibers. Therefore, the parasympathetic nerve activity was indirectly expressed by variability of the heart rate as the consequence of the sinus node activity in most research trials [39].

Interestingly, an attempt at autonomic system blockade by injection of propranolol and atropine in bradycardic athletes results in the persistence of bradycardia in examined individuals [42]. This finding may lead to the conclusion that resting bradycardia may actually be the consequence of intrinsic heart changes [43].

## 11. Non-Autonomic Regulation 

Recent data showed that high vagal tone could not fully explain training-induced bradycardia [44]. A brief review of the current literature associates bradycardia with a change in the intrinsic activity of the sinus node or AV node [45, 46, 47]. Also, low heart rate may be related to dysfunction of the sinus node or AV node secondary to structural heart disease such as infarction, cardiomyopathy, genetic disorders or infiltrative disease for instance sarcoidosis, amyloidosis and haemochromatosis. Recent evidence highly associates low heart rate adaptation with molecular alterations in the sinus node or AV node [47, 48]. The molecular basis of bradycardia indicates that remodeling of ion channels of the sinus node or AV node plays a key role in determining heart rate [43, 47]. A collaborative study by Alicia D’Souza *et al*. [39] gave the evidence that resting bradycardia is the result of downregulation of hyperpolarization-activated, cyclic nucleotide-gated 4 (HCN4) and the hyperpolarization-activated cation current, known as funny current. HCN4, which is localized in pacemaker cells of the sinoatrial node (SAN) is mostly involved in diastolic depolarisation which means it is mainly responsible for conducting the action potential from the SAN to the surrounding atrial muscle [49, 50, 51]. Downregulation of HCN4 was attributed to training-induced changes of transcriptional regulators - downregulation of T-box 3, upregulation of neuron-restrictive silencer factor (NRSF) and microRNA-1 [48]. Furthermore, several mutations of HCN4 lead to a more frequent appearance of sinus bradycardia and/or more complex rhythm disturbances [50].

## 12. Differences in the Diversity of Sport Modalities 

The investigations of recent studies demonstrated that the modality of sports may suggest the underlying mechanism of bradycardia. Azevedo *et al*. [8] reported that according to their research professional runners are more likely to have more pronounced resting bradycardia than cyclists. Their observation shows that reduced heart rate in runners is mainly associated with a predominant vagal effect on autonomic regulation, while cyclists’ bradycardia depends on structural heart remodeling which is eccentric and concentric hypertrophy [8]. A similar finding for hypertrophy as the responsibility for heart rate reduction was acquired by Zakynthinos *et al*. [10] who examined electrocardiographic features of bradycardia in water-polo athletes and Kaur [9] who investigated wrestlers athletes.

Recent evidence indicates that low heart rate in athletes can vary depending on the type of exercise training. Studies observed that a heart rate of about 50–60 bpm is generally assumed to be achieved during endurance activity such as long-distance running. Discussing the representatives of different types of sports, 50 to 90% of them reached a heart rate of 50 bpm. However, a relatively low heart rate in the range of 30–40 bpm has been particularly associated with professional cyclists [52].

## 13. The Long-Term Follow-up and Side Effects—Is it all Worth it?! 

It may be hypothesized that bradycardia can lead to symptoms ranging from fatigue, dyspnea, dizziness to syncope and arrhythmias more profoundly among bradycardic endurance athletes [53]. However, the prevalence of these symptoms is not more frequent in this group [54]. Matelot *et al*. [54] revealed that athletes exhibiting deep bradycardia are not more prone to reflex syncope and arrhythmias than their non-bradycardic peers.

Furthermore, although recent studies revealed that chronic bradycardia persists in the majority of post-retirement athletes, the reduced heart rate was not significantly associated with syncope, palpitations or dizziness. Interestingly, the resting bradycardia was linked to the regularity of exercise and duration in years of intensive training [55].

## 14. Management of Bradycardia in Athletes 

The effective handling of bradycardia in athletes poses an ongoing challenge, owing to the intricate and not fully elucidated nature of its pathophysiology [56]. According to the European Society of Cardiology (ESC) recommendations, pacemaker implantation for sinus node dysfunction (SND) is advised primarily for symptomatic patients, where bradycardia leads to symptoms like fatigue, dizziness, fainting, or shortness of breath [57]. Asymptomatic sinus disturbances (e.g., sinus bradycardia or sinus pauses) are common in athletes due to physiological adaptations to exercise. These changes are typically benign but sometimes may cause symptoms like those mentioned above, warranting a reassessment of sports involvement. Typically, asymptomatic pauses (<3 seconds) are not concerning, but longer pauses, especially with symptoms requiring further tests like ECGs, 24-hour monitoring, and exercise tests. For athletes experiencing symptoms related to sinus bradycardia or pauses, a break from sports activity often leads to symptom resolution within a month or two. Rarely, in cases where symptoms persist or bradycardia is unresponsive to other measures, a permanent pacemaker may be required, though this is exceedingly rare among athletes [58, 59].

Regarding AVBs, the ESC recommends pacing for all third-degree AVB cases, second-degree Type II AVB regardless of symptoms, symptomatic second-degree Type I AVB and first-degree AVB with marked PR interval prolongation (i.e., ≥300 ms) due to the risk of severe symptoms or possible progression [57]. Among athletes, asymptomatic first-degree AVB or second-degree Type I AVB is frequently prevalent and usually does not restrict sports participation. These disturbances, considered benign features, stem from increased parasympathetic stimulation. Clinical trials provide evidence that reflex sympathetic maneuvers (e.g., Valsalva maneuver) immediately improve A-V (atrioventricular) conduction, and complete normalization may be obtained with the administration of sympathomimetic (e.g., isoproterenol) and anticholinergic (e.g., atropine) drugs [60]. Moreover, first-degree and second-degree type I AVB tend to resolve during exercise, and even if symptoms occur, temporary cessation of sports followed by re-evaluation suffices in most cases.

In healthy sportsmen with vagal nerve overstimulation, asymptomatic second-degree AVB type 1 and type 2 do not require particular management, but recommendations for decreased exercise load should be given.

**Fig. 3.**
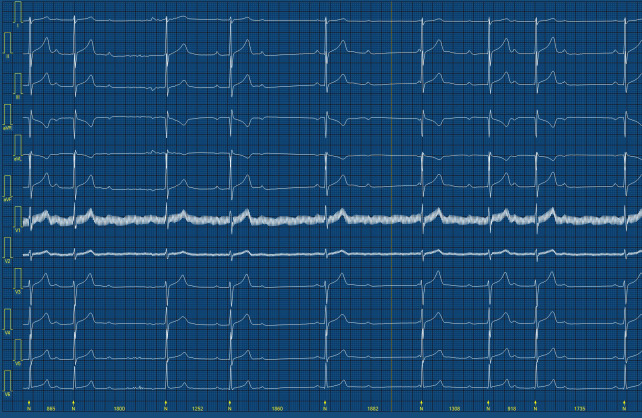
**Example of second-degree type 1 atrioventricular block resembling high grade atrioventricular block**.

The incidence of second-degree type II AVB in athletes is rare, yet when it does occur, it is often prone to be misinterpreted as second-degree type I AVB. Distinguishing between these types relies on their electrocardiographic features but it can be particularly challenging, especially in cases with unusual presentations. In Fig. 3 the authors present a nighttime clipping of 24-hour Holter monitoring with second-degree type 1 AVB and escape junctional beats mimicking high grade AV block. The primary source of confusion stems from the irregular PR sequences (intervals between atrial and ventricular excitations). Hence, strict adherence to clear definitions and a nuanced understanding of the disparities between type I and type II second-degree AVB is vital, particularly when athletes show no symptoms. This precision is crucial to avoid diagnostic errors and therapeutic doubts regarding pacing [61].

Clipping from 24-hour Holter monitoring with second-degree type 1 atrioventricular block and escape junctional beats leading to heart’s rhythm irregularity and mimicking high grade atrioventricular block.

Of note, while engaging in exercise usually overcomes first and second-degree type 1 AVB leading to increased heart rate according to exercise load, physical workout may also rarely exacerbate or trigger second-degree type 2 AVB [62]. In certain elite athletes, second-degree type 2 AVB and occasionally third-degree AVB episodes may manifest during the night-time (24-hour Holter monitoring). However, if these are evident on a standard ECG during the day, a thorough evaluation is necessary to eliminate the possibility of structural heart disease or other underlying conditions, particularly if the disorder persists during physical exertion [63]. In patients with intraventricular conduction disease or AVB of unknown level, exercise testing may be considered to disclose advanced infranodal atrioventricular block which carries a high risk of progression to complete heart block and warrants pacing even in the absence of symptoms [57].

In patients with structural heart disease overlapping sports activity a pacemaker might be suggested for individuals with symptomatic second-degree AVB type 1, while in second-degree AVB type 2 or third-degree AVB regardless of symptoms, according to current guidelines [58, 59].

## 15. Can an Athlete Play with a Pacemaker?

Athletes with an implanted pacemaker and heart disease may only engage in sports that align with their heart condition’s limitations [58]. Individuals entirely pacemaker-dependent should avoid sports involving potential collisions that could harm the pacemaker system, while those who are not pacemaker-dependent may engage in collision-prone sports if they acknowledge and consent to the risk of pacemaker damage [59]. Sports involving physical collisions or bodily contact may lead to damage to the electrodes or even skin perforation where the pacing unit is implanted so wearing appropriate protective equipment and padding is necessary. Significant arm movements could also heighten the chance of later pacemaker damage due to subclavian compression (so-called “subclavian crush syndrome”), potentially leading to insulation or conductor failure, therefore depending on arm dominance and the nature of sports activities, the pacing device should be implanted on either the right or left side [64]. Moreover, a can of pacemaker could be placed submuscularly (and not typically subcutaneously) in order to reduce the risk of mechanical injury. Furthermore, modern pacemakers are usually resistant to electromagnetic interference, but it is crucial to carefully evaluate the potential impact of presumed interfering sources in the athletes’ environment, e.g., electronic starting gates or electronic scoring equipment. Any interference could disrupt pacing temporarily, which is a major concern for pacemaker-dependent athletes, requiring increased caution in those individuals [58].

## 16. Conclusions

Athletes, particularly those engaging in endurance sports, often exhibit a wide spectrum of tachy- and bradyarrhythmias, posing a unique challenge for clinicians and researchers. Arrhythmias with their burdensome symptoms can lower the quality of life and lead to dangerous consequences. Balancing the management of arrhythmias with the preservation of athletic performance is a demanding task for both patients and clinicians. Clinicians, prioritizing optimal disease management based on guidelines for the general population, may inadvertently overlook the unique priorities of athletes, leading to tensions between patients and healthcare providers. Limited knowledge in handling athletes with tachy- and bradyarrhythmias, a lack of understanding of athletes’ priorities, and the absence of educational resources specifically tailored for this patient group can contribute to the above-mentioned problems, necessitating a comprehensive approach to this kind of patient [35, 65]. All in all, one should conclude: run Forest, run with common sense!
